# Nucleotide-Induced Nanoscale Changes in the Mechanical Properties of Rat Cerebellar Astrocytes: Selective Stimulation and Blocking of the Purinergic Receptor P2X7 †

**DOI:** 10.3390/ijms231911927

**Published:** 2022-10-07

**Authors:** Juan Carlos Gil-Redondo, Jagoba Iturri, Yaiza Trueba, María Benito-León, Raquel Pérez-Sen, Esmerilda G. Delicado, José Luis Toca-Herrera, Felipe Ortega

**Affiliations:** 1 Department of Nanobiotechnology (DNBT), Institute of Biophysics, BOKU University for Natural Resources and Life Sciences, Muthgasse 11 (Simon Zeisel Haus), A-1190 Vienna, Austria; 2 Sección Departamental de Bioquímica y Biología Molecular, Facultad de Veterinaria, Instituto Universitario de Investigación en Neuroquímica (IUIN), Instituto de Investigación Sanitaria del Hospital Clínico San Carlos (IdiSSC), Universidad Complutense Madrid, 28040 Madrid, Spain

**Keywords:** astrocytes, P2X7 nucleotide receptor, force spectroscopy, force mapping, time-lapse video-microscopy

## Abstract

As members of the family of nucleotide receptors, P2X7 receptors are of particular interest due to their unique structural and pharmacological characteristics. As ATP-gated ionic channels, P2X7 receptors in their activation elicit membrane depolarization; extracellular calcium influx; and activation of several downstream intracellular signaling pathways, some of them independent of the ionic channel activity. Further interactions of P2X7 receptors and cytoskeleton-related proteins have also been confirmed, and we previously described the effects of P2X7 receptor stimulation on the morphology of rat cerebellar astrocytes. In the present work, we used time-lapse video microscopy and atomic force microscopy (AFM) to elucidate the effects of P2X7 receptor stimulation on the morphology, migratory capabilities, and mechanical properties of rat cerebellar astrocytes in vitro. Stimulation of P2X7 receptors with the selective agonist BzATP specifically caused an increase in cell size, motility, and number of membrane protrusions of the astrocytes in culture. These effects were reverted when cells were previously treated with the competitive antagonist of P2X7R, A 438079. AFM analysis also showed an increase in cell stiffness and viscosity after P2X7 receptor stimulation. Surprisingly, these effects on the mechanical properties of the cell were not blocked by the treatment with the antagonist. Fluorescence microscopy analysis of the actin cytoskeleton showed an increase in actin stress fibers after BzATP treatment, an effect that again was not blocked by previous treatment with the antagonist, further confirming that the effects of P2X7 receptors on the cytoskeleton of astrocytes are, at least in part, independent of the ionic channel activity.

## 1. Introduction

Nucleotides act as extracellular messengers that regulate vast functions in almost any cell type present in animal organisms [1], and their effects are particularly important in the nervous system, both in physiological and pathological conditions [2,3]. On cells, nucleotide actions are mediated by P2 receptors, a family of membrane receptors that can be further classified into two different groups: P2Y and P2X receptors [4,5]. From these, P2YRs are metabotropic receptors that belong to the G-protein-coupled receptor (GPCR) superfamily. They are formed by seven-membrane-spanning domain proteins coupled to G proteins that are activated selectively by adenosine and uridine tri- and diphosphates. In total, eight different subtypes have been discovered in mammalians: P2Y_1, 2, 4, 6, 11, 12, 13,_ and _14_ [4]. P2XRs, in turn, are ATP-gated ionic channels consisting of seven distinct subunits (P2X1-7) that are assembled in homotrimers or heterotrimers. The trimeric assembly of the subunits constitutes the functional receptor, whose structure consists of a chalice-like extracellular domain where the three ATP-binding sites are found: the ionic pore, composed by six transmembrane (TM) domains, two TMs for each subunit, and a shorter intracellular domain. Upon nucleotide activation, P2XRs have been shown to elicit membrane depolarization, extracellular calcium influx, and activation of several intracellular signaling pathways, both in a calcium-dependent and -independent manner [5].

Among the different P2XRs described, the P2X7 receptor is of particular interest for our studies because of its unique structural and pharmacological characteristics. P2X7R is a homotrimer whose subunits show the longest C-termini of all P2X subunits (200 additional amino acids), and its affinity for ATP is 100 times lower than that shown by the other P2X receptors [5,6]. Besides its well-known role in the regulation of the immune response [7,8] (it was previously known as the P2Z receptor from macrophages and lymphocytes [9]), P2X7R is implicated in important physiological and pathological processes in the central nervous system, including the modulation of neurotransmitter release, neuritogenesis and synapse formation, the neuroprotective role in cerebellar granule neurons and astrocytes, and several neurodegenerative diseases [6]. Likewise, multiple intracellular signaling pathways are regulated by P2X7Rs. For instance, P2X7R is known to activate several phospholipases, protein kinases (protein kinases C and D, mitogen-activated protein kinases, and the cellular sarcoma tyrosine kinase), and the phosphoinositide 3-kinase/Akt pathway [10]. P2X7 receptor is also known to interact with several cytoskeleton-related proteins, such as the extracellular matrix protein laminin-α3, integrin β2, α-actinin, and β-actin [11], and it has also been reported that P2X7R stimulation in macrophages causes actin-cortex reorganization that is required for the membrane blebbing [12]. Hence, such tight interaction between P2X7Rs and the cytoskeleton, upon external stimuli, ultimately implies their involvement onto changes in the mechanical properties of the cell. Indeed, previous research in this regard indicated that the signaling of these ionotropic receptors in rat cerebellar astrocytes also includes metabotropic pathways independent from the ionic channel activity, such as MAP kinases ERK1/2 and protein kinase D activation [13,14,15]. Interestingly, while activation of ERK1/2 kinases by P2Y_2_/P2Y_4_ receptors induce migration of the astrocytes [16], activation of these kinases by P2X7 receptors cause complex changes in cell morphology [17]. Therefore, P2X7-originated changes in the cytoskeleton and, by extension, in the mechanical properties of the cell are to be expected. However, a detailed quantification of the triggered mechanical variation has not been reported so far.

**Figure 1:**
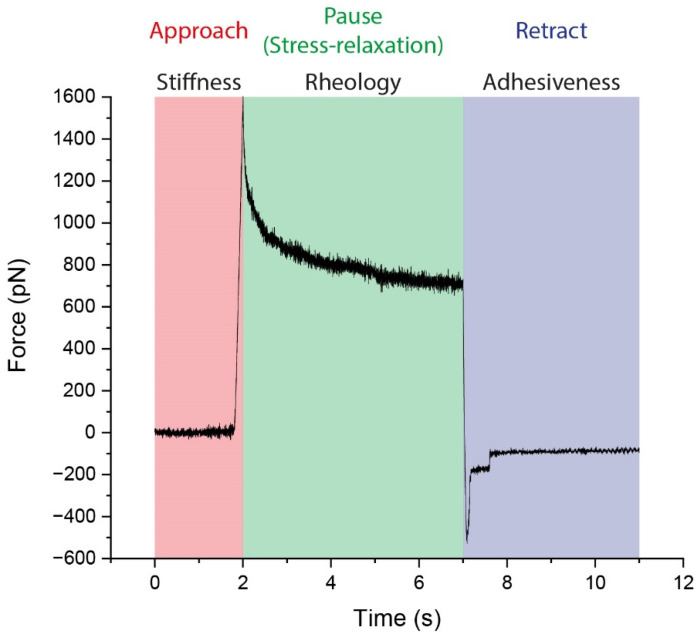
Force vs. time curve. Colored scheme showing the mechanical information derived from each of the different segments in one experiment: elastic modulus and stiffness (from the initial deformation and the slope, subsequently), stress relaxation (by fitting the decay obtained when fixing the Z position of the indenting tip during the pause time it is possible to elucidate the relaxation time), and adhesion-related factors (the minimum of the peak indicates the maximum adhesive force, and the integration of the area under the curve relates to the adhesion work).

In this regard, atomic force microscopy (AFM) is one of the most extended techniques employed to study the mechanical properties of cells and biomaterials. This technique, mostly known for its application on sample topography imaging, can also be used to analyze changes in the stiffness, rheology, and adhesive properties of cells, and it is based on the perpendicular indentation of the cell membrane with a tip located at the end of a cantilever [18,19,20,21]. The forces resulting from the controlled interaction between the tip and the sample are displayed as force-vs.-distance or force-vs.-time curves (see Figure 1). Initially, the tip is approached to the sample, and the indentation of the membrane provides information about the deformability and stiffness of the cell [19]. Upon reaching a previously stablished maximum loading force value (or setpoint), the tip can be maintained at a certain height (stress–relaxation experiments) or can be pushed further in order to keep constant the force applied (creep experiments). Both experimental situations correlate with the rheological properties of the cell and provide quantitative information over factors such as relaxation times and viscosity [18]. In a last step, the tip is retracted from the sample, causing the cantilever to deflect in the opposite direction due to the adhesion between cell membrane and tip, thus delivering information about the adhesive properties of the cell, such as the maximum adhesive force, the adhesive work required to split the tip-cell contact, and to identify the potential formation of membrane tethers during retracting motion [20,22,23,24,25].

On the basis of these principles, previous collaborative work between our groups showed how nucleotide receptor regulation can influence the mechanical properties of cells at the nanoscale, as concluded from the studies performed on P2YR-expressing endothelial HUVEC cells and rat cerebellar astrocytes [26]. Stimulation of P2Y_2_Rs with UTP in both cell types caused an increase in cell stiffness and a decrease in adhesive properties. Further experiments with astrocytes showed that the effects of the purinergic agonist 2MeSADP on the mechanical properties of the cell were mediated by P2Y_13_ receptors and negatively modulated by P2Y_1_ receptors. As a logical continuation to those initial studies, and attending to what is described above, unravelling the role of P2X7 nucleotide receptors on the mechanical properties of rat cerebellar astrocytes becomes extremely interesting.

Following this, in the present work, we show how stimulation of the P2X7 receptor of rat cerebellar astrocytes with the selective agonist BzATP specifically induced an increase in the cell size, motility, and number of membrane protrusions of the astrocytes in culture. Such effects were reverted when cells were treated with the competitive antagonist of P2X7R, A 438079 (henceforth referred to as A43), prior to treatment with the agonist BzATP. Interestingly, when AFM was employed to further disentangle the role of P2X7 receptor in the modulation of the mechanical properties of this population on the nanoscale, an increase in the stiffness of the cells was observed. However, treatment with A43 did not effectively block such increase in stiffness caused by the agonist, pointing to the fact that the effects observed were not exclusively dependent on the ionotropic activity of the receptor but also on additional effects caused by independent metabotropic pathways that remained unblocked. Similar results were obtained when analyzing the viscous moduli of astrocytes, which indicated potential changes occurring in the cytoskeleton. In order to obtain a more general view on the induced effects, complementary force spectroscopy maps performed over the whole body of treated/untreated cells allowed for the observation of higher stiffness values and stronger adhesive forces throughout all the cell body after treatment with the agonist, further supporting the evidence of changes on the cytoskeleton level.

## 2. Results

### 2.1. Stimulation with BzATP Modified the Cell Size and Migration of Rat Cerebellar Astrocytes In Vitro

**Figure 2:**
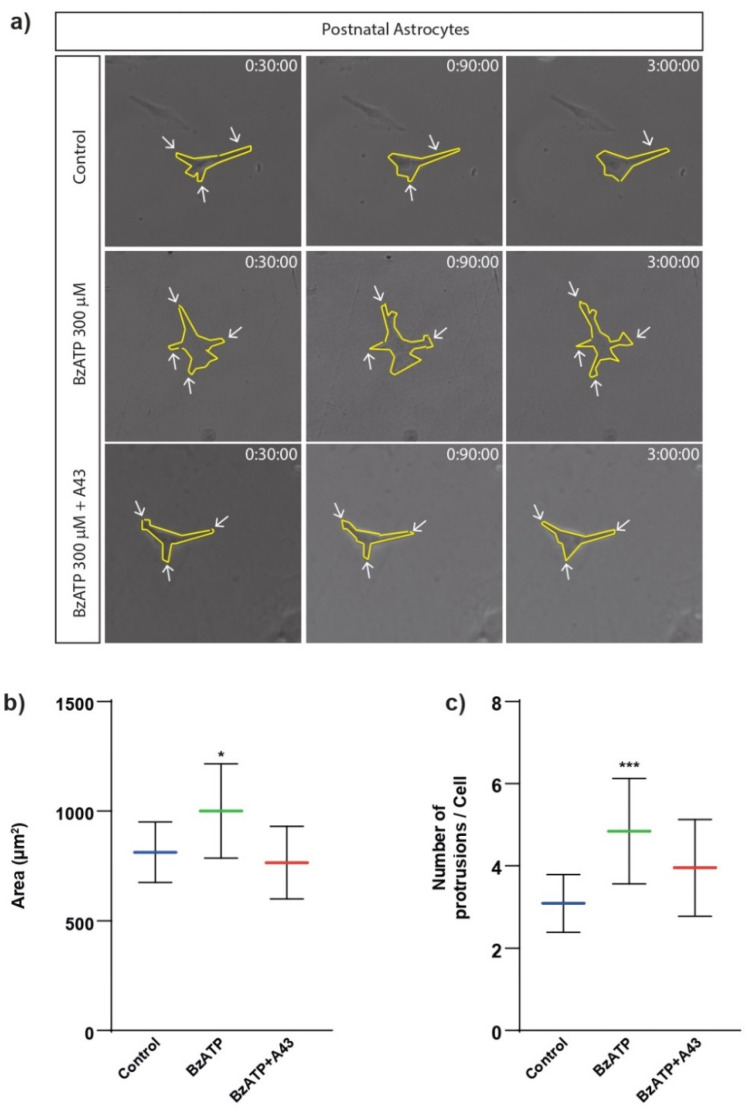
Effects of BzATP treatment on cell size and membrane protrusions of rat cerebellar astrocytes. (**a**) Bright field images of astrocytes obtained after 3 h of time-lapse video microscopy. (**b**) Histogram showing the evolution of the cell size (area, in µm^2^), during a period of 3 h, in basal conditions or in the presence of 300 µM BzATP. The effect of the P2X7 antagonist A43 is also included. In this case, astrocytes were preincubated with 10 µM A43 for 5 min, prior to BzATP addition. (**c**) Histogram indicates the number of membrane protrusions present in astrocytic cells across the imaging experiment. Whiskers indicate standard deviation (SD); statistically significant differences were calculated by employing a one-way ANOVA with Dunnett’s post-test and are indicated by * when *p* < 0.05 or *** *p* < 0.001. Experiments with only A43 are not considered for comparison because of the lack of visual evidence.

It was previously shown how the stimulation of metabotropic P2Y_13_ receptor was sufficient to modify the cell motility of postnatal cerebellar astrocytes in primary cell cultures [26]. To explore whether the activation of the ionotropic P2X7 receptor may also induce changes in the cell size and migration of this cell population, cerebellar astrocytes were incubated in the presence or absence of the selective agonist of the P2X7R, BzATP (300 µM), for 1 h. Astrocytes were subsequently placed on a time-lapse video microscopy device and monitored for 3 h, acquiring bright field images every 5 min. These experiments allowed us to measure the cell size (area in µm^2^) and the distance covered by the astrocytes in the plate culture. As shown in Figure 2 and the values in Table 1, the treatment with BzATP resulted in a significant increase in the cell size of astrocytes (1001 µm^2^ in treated cells versus 812 µm^2^ in control cells, at 20%). Importantly, this change was avoided in the presence of the P2X7R antagonist A43 (10 µM), pointing to the P2X7 receptor as the responsible for the effects observed. Cell size of astrocytes pretreated with A43 and BzATP was 765 µm^2^. The number of membrane protrusions in the isolated astrocytes was also measured. While untreated astrocytes exhibited 3.10 protrusions/cell, cell treatment with BzATP increased the number of protrusions (4.85). Moreover, the P2X7R antagonist A43 also reduced the BzATP effect (3.96) (Figure 2a,c).

**Figure 3:**
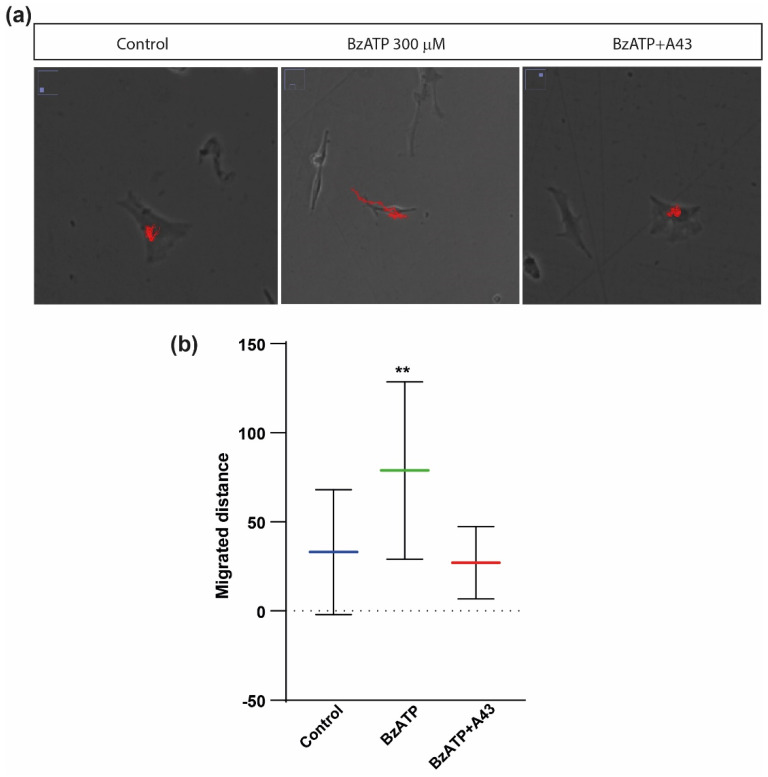
Effects of BzATP treatment on migratory capacity of rat cerebellar astrocytes. (**a**) Bright field images of astrocytes obtained after 3 h of time-lapse video microscopy in control astrocytes and astrocytes treated with 300 µM BzATP in the presence or absence of the antagonist A43. (**b**) Histogram showing the distance travelled in pixels by the astrocytes during a period of 3 h, in basal conditions, or after incubation with BzATP for one hour, in the presence or absence of antagonist A43. Whiskers indicate SD; statistically significant differences were calculated by employing a one-way ANOVA with Dunnett’s post-test and are indicated by ** when *p* < 0.01.

Next, we tested whether cell migration was also affected by the stimulation of the P2X7 receptor, assessed by measuring the distance covered by the astrocytes throughout the imaging experiments. As shown in Figure 3 and Table 1, BzATP induced a significant increase in the distance covered by individual astrocytes. Similar to what was observed for cell size measurements, the effect on cell motility was also blocked by the presence of A43, further confirming the involvement of the P2X7 receptor in the increase in astrocytic migration capacity.

Table 1 summarizes data obtained for cell size, number of membrane protrusions, and cell migration of astrocytes after the different treatments by the time-lapse microscopy approach. A colored box is used to attract the reader’s attention onto the reversion of the effects of BzATP by the P2X7R antagonist (A43).

The implication of these observed effects is that P2X7R stimulation generates a dynamic response in cells, with either their shape or motility being certainly affected. Therefore, and due to the nature of this response, it is somehow evident that the mechanical properties of the astrocytes undergo a re-adjustment during this process. From mere visual analysis, such readjustment cannot be appropriately quantified, and the need for a complementary technique is highlighted.

### 2.2. Mechanical Response of Astrocytes upon P2X7 Receptor Stimulation/Blocking: Elastic Modulus

**Figure 4:**
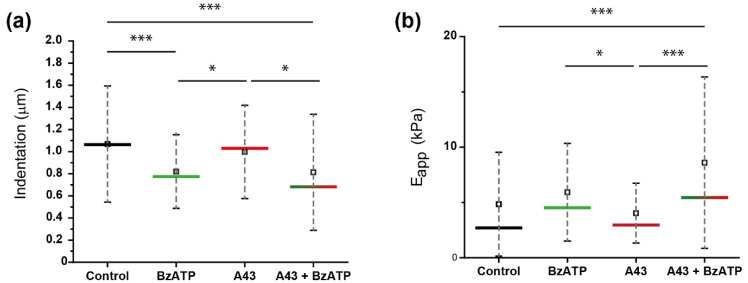
Cerebellar astrocyte nucleotide stimulation influence on approach-derived mechanical factors. (**a**) Cell indentation before maximum load (1.5 nN) achievement and (**b**) calculated apparent elastic moduli (E_app_) variation for untreated cells (control, black), and cells exposed to BzATP agonist (green), A43 antagonist (red), or both A43 + BzATP (gradient). The horizontal line indicates the median, and the square refers to the mean value, which are shown numerically in Table 2. Whiskers indicate SD. Statistically relevant variations *p* < 0.05, and *p* < 0.001 are highlighted by *, and ***, respectively.

To solve the aforementioned issue, the mechanical response of rat cerebellar astrocytes upon treatment (for 1 h) with BzATP (300 µM), either in the presence or absence of the P2X7R antagonist A43, was determined by means of AFM indentation experiments. Figure 4 and Table 2 indicate the cell indentation required to reach a pre-defined loading force of 1.5 nN and the correlated apparent Young’s modulus values (E_app_) for the respective astrocytes after the different treatments (Table S1 in the Supplementary Materials section shows further calculated parameters).

As one might intuitively predict, larger indentations connect to a softer material being probed. At first glimpse, and compared to untreated astrocytes, an increase in the stiffness (E_app_) of the astrocytes after nucleotide stimulation of P2X7 receptors with BzATP can be observed (4.84 ± 4.69 kPa in control conditions vs. 5.93 ± 4.42 kPa after P2X7 stimulation), while initial treatment with the antagonist compound (A43) did not cause any significant effect in these same factors, and cells resembled those used as control (untreated). Interestingly, subsequent exposure of A43 treated cells to BzATP (A43 + BzATP) also caused a significant increase in the stiffness of the cells (8.60 ± 7.76 kPa), therefore indicating that the intended blocking by A43 was ineffective.. A similar behavior was described for pre-blocked P2YRs treated with 2MeSADP, which was finally connected to the intervention of two distinguishable contributors (P2Y_1_ and P2Y_13_) [26]. Then, and attending to our current observation, the intervention of a second element, which is not suppressed by action of the A43, can also be envisaged.

Table 2 shows the values for both indentation (in µm) and apparent elastic modulus (E_app_, kPa) as calculated from the respective agonist–antagonist incubation processes. A colored box was employed to attract the reader’s attention onto the variation caused by combined blocking/stimulation with respect to the individual treatments or the situation without a pre-blocking of the P2X7Rs (which stayed at 35% lower).

These values confirm the lack of mechanical response observed for cells treated with the antagonist A43, which was statistically confirmed as non-significant (Figure 4) in comparison with control cells, meaning that such a treatment leaves the cells in an intact (at least structurally) status before the action of BzATP on them. Therefore, a contributive effect from that side to the large boost observed afterwards can be discarded, only being assigned to selective modulation of the membrane receptors.

### 2.3. Stress Relaxation and Viscous Properties of Astrocytes

In addition to the analysis performed on the approach segment, the variation in the viscoelastic properties of the astrocytes resulting from P2X7R stimulation was determined from the stress–relaxation profiles obtained during the 5 s pause in tip-cell contact, at constant height, after a 1.5 nN load was reached. Such stress relaxation is inherent to viscous materials, and in the case of cells, it is tightly connected to the cytoskeleton activity and the overall rheological properties. Thus, a double exponential decay was fitted to the force-vs.-time profiles (see description in Figure 1) on which a Zener model of the cell was applied to determine different viscoelastic parameters. Such a model considers the cell as a viscoelastic material comprised by one spring and two Maxwell elements (a spring and a dashpot in series) set in parallel. The viscoelastic parameters determined by this cell model are the equilibrium modulus ($E_{\infty }$); the elastic (compressive) moduli of the springs of both Maxwell elements ($E_{1}$ and $E_{2}$); the instantaneous modulus ($E_{inst}$); and, in addition, the relaxation times ($\tau_{1}$ and $\tau_{2}$) and viscosities ($\eta_{1}$ and $\eta_{2}$) of the dashpots of both Maxwell elements. Effects of the different treatments in the values of all the parameters can be found in Tables S2 and S3. In general, treatment with BzATP caused an increase in the compressive moduli, an effect that, once again, was not blocked, but seemed to be further enhanced by the pre-treatment with the antagonist A43 (see values for the equilibrium and instantaneous moduli).

**Figure 5:**
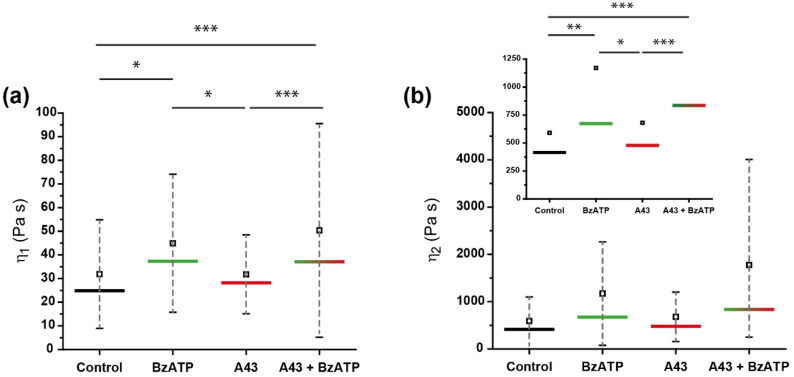
Calculated viscous moduli 1 (**a**) and 2 (**b**) for rat cerebellar astrocytes in control conditions (black line), or those treated with BzATP (green), A43 (red), or both (gradient). The horizontal line indicates the median, and the square refers to the mean value, which are shown numerically in Table 3. Whiskers indicate SD. Inset in (**b**) shows the same values in a shorter scale in order to appreciate the differences in the mean and the median. Statistically relevant variations *p* < 0.05, *p* < 0.01, and *p* < 0.001 are highlighted by *, **, and ***, respectively.

The relaxation times calculated for the viscous dashpots of the Maxwell elements were around one-tenth of a second and two seconds for the first ($\tau_{1}$) and the second elements ($\tau_{2}$), respectively (Table S3). These values were close to those observed in previous works where this model of the cell was used [27,28]. Indeed, the work by Moreno-Flores et al. (2010) demonstrated the implication of actin cytoskeleton in both the relaxation times and viscosity of Maxwell elements and, on the basis of previous studies [29], associated the first Maxwell element with the membrane-actin cortex and the second element with the cytoskeleton response. In our case, although the trend of those relaxation times did not appear to be significantly affected by the different treatments (except for a certain trend in $\tau_{2}$), the viscosity moduli calculated for both elements did, and such a response mainly occurred after the corresponding treatment with BzATP (Figure 5). As observed for the rest of the parameters, the antagonist A43 did not effectively block the increase in viscosity caused by exposure to the agonist but further promoted such an increment. This potentiation, although not statistically significant, was more pronounced in the case of the second Maxwell element (Figure 5b). An additional effect brought about by such receptor passivation was the much broader variability of the values obtained, most likely attributed to the diverse number of receptors expressed between the indented cells (*n* > 30).

A more accurate comparison than that from mere visual observation of the trends in Figure 5 can be established from the values collected in Table 3 for the viscosities of both Maxwell elements, $\eta_{1}$ and $\eta_{2}$. While the former yielded an increase of around 10–15%, between the direct and the pre-blocked exposure of P2X7 to BzATP, the later reached up to the 50% increase (1171.2 Pa s vs. 1775.0 Pa s). Indeed, this value represents a threefold viscosity increase in comparison to untreated (control) astrocytes. Since the $\eta_{2}$ term can be approximated to the potential cytoskeletal contribution to the overall viscoelastic variation, as explained above, then the combined blocking/stimulation treatment of purinergic P2X7 receptors refers to a critical transformation or rearrangement induced at the inner structural level (i.e., cytoskeletal fibers/filaments).

### 2.4. Force Mapping on Individual Cells: Mechanical Factor Distribution

An alternative measuring mode to regular force spectroscopy (explained in the sections above) is brought about by performance of whole-body cell mappings (force volume measurements), which enable the characterization of the overall distribution of the cytomechanics-related factors. This methodology becomes a very interesting approach to the receptor stimulation hypothesis under analysis. As main drawback, one should cite the noticeable loss in data precision in comparison with single-point indentations applied in the measurements above (allowing for a better controlled statistical analysis, more representative of the mechanical response of the cell), the larger experimental time required, and the logical distribution variation between cells (even under the same treatment) according to the respective receptor expression. Despite these weak points, mapping studies were here applied for the first time on individual cerebellar astrocytes in order to explore alternative routes for addressing the cell response to external stimulation of P2X7 nucleotide receptors. Similar studies have been already reported in the literature for sliced brain (pituitary gland) tissue to show the spatial distribution of its elastic properties [30], from which the authors described the non-uniformity and gradient-like appearance of their values. The information thus obtained (colored 2D distribution maps) provides a logical extension to the single point experiments and can be considered an approximate intermediate level between the measurements in the previous section and the dynamic micrometric analysis of living cells by microscopy (i.e., motion, area variation). Hence, the output might render a lower statistical accuracy but, simultaneously, generates a more general view on the spatial response of cells to P2X7 receptor stimulation.

**Figure 6:**
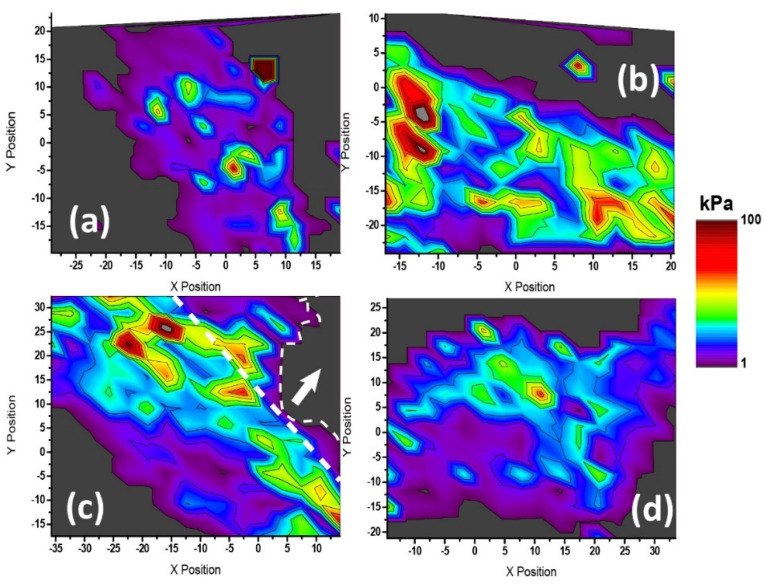
Cell mapping on elastic (Young’s) modulus variation resulting from P2X7R stimulation of rat cerebellar astrocytes. (**a**) Control (untreated), (**b**) BzATP, (**c**) A43, and (**d**) A43/BzATP. The thick dashed line in (**c**) delimits two neighboring astrocytes. The inner thin line and the arrow point at the area occupied by the body region of such an attached cell, from which values were removed to avoid a distorted interpretation. The color scale bar ranges in all cases between 1 and 100 kPa. Out-of-range values are shown in dark grey (i.e., glass substrate).

Figure 6 shows the 2D mapping of the elastic moduli extracted from the four systems under comparison: (a) untreated, (b) BzATP-treated, (c) A43-treated, (d) A43 + BzATP-treated. At first glimpse, the overall distribution of the values appeared quite affected when comparing that in Figure 6a to any other conditions (Figure 6a–d). In an untreated astrocyte, the highest elastic modulus values recorded appeared very localized into central positions, while the rest of the body yielded very regular and homogeneous values. This behavior was significantly affected when receptor stimulation took place. For these cases, larger moduli were observed all over the area being tested, with a notorious increment of cyan to green colors (in the range of 20–30 KPa). It must be noted that the presence of sharp local spots with very large values (such as in (b) and (c)) could be explained by underlying substrate detection, with astrocytes being very thin, while nucleus-topping regions appeared as broader and gradually incrementing (i.e., Figure 6d). The aligned stiffening observed in Figure 6c resulted from the presence of a neighboring cell, which caused the reinforcement along the cell–cell contact. When those values were taken aside, the behavior was a hybrid between Figure 6a,d. A quantitative comparison between the respective values is presented in the shape of histograms (see Figure S1).

**Figure 7:**
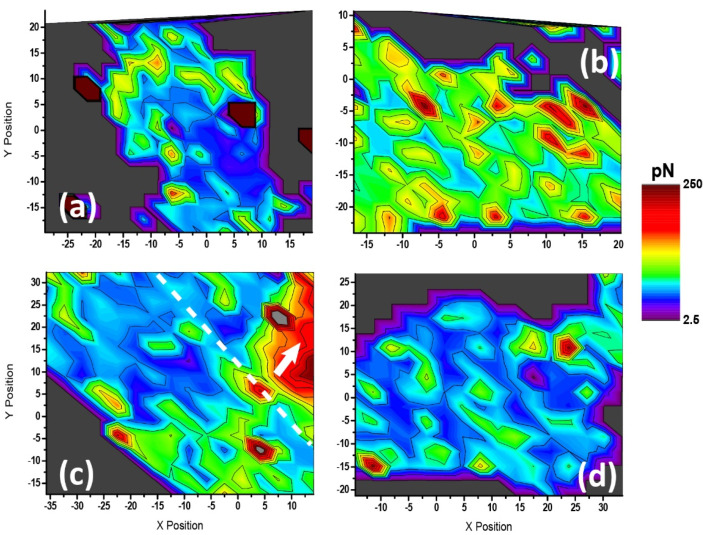
Cell mapping on adhesion force variation resulting from P2X7R stimulation of rat cerebellar astrocytes. (**a**) Control (untreated), (**b**) BzATP, (**c**) A43, and (**d**) A43/BzATP. The thick dashed line in (**c**) delimits two neighboring astrocytes. The inner thin line and the arrow point at the area occupied by the body region of such an attached cell. The color scale bar ranges in all cases between 2.5 and 250 pN (piconewton). Out-of-range values are shown in dark grey (i.e., glass substrate).

Similar results were obtained for the maximum adhesion force values (from the peak minimum in the retraction segment), which is another factor that correlates to the variations in the cell state caused by the different stimulations of the P2X7 receptor (Figure 7). Here, untreated cells presented larger values localized closer to the edge, while the central part of the body remained less prone to attachment to the AFM tip. In the other three cases, distribution of the values was, by far, more homogeneous, being very surprising the overall increment observed for adhesion values upon exposure to BzATP (without previous receptor blocking).

The use of A43 shown in both Figure 7c,d seemed to slightly attenuate such a huge response in terms of absolute values, but still the distribution was affected to a large extent. In any case, these results over the whole body were in close connection to values calculated for one single point indentation of astrocytes (Table S1). In those, direct exposure of control cells to BzATP unexpectedly increased the mean maximum adhesion force calculated (194.9 to 213.2 pN), while treatment with A43 and subsequent BzATP exposure followed an opposite trend (176.8 to 142.0 pN). A quantitative comparison between the respective values is presented in the shape of histograms (see Figure S2).

### 2.5. Spatial Reorganization of Actin Cytoskeleton: Phalloidin Staining

**Figure 8:**
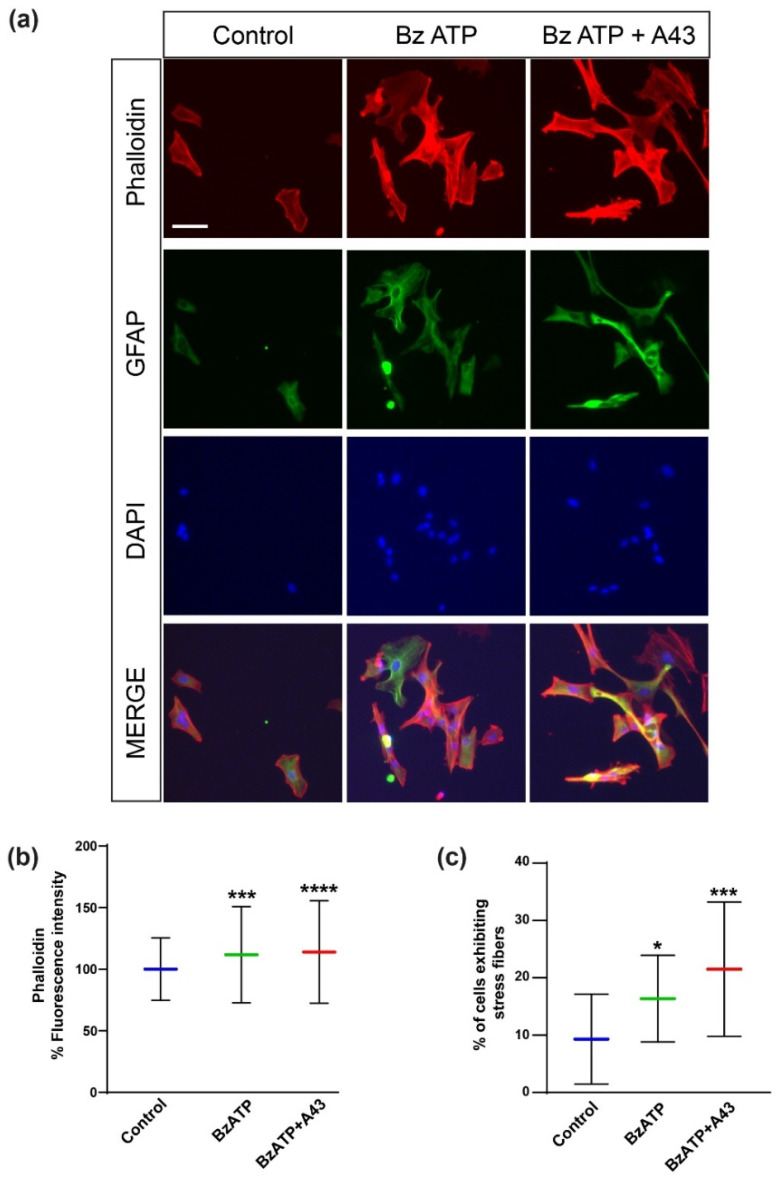
P2X7 receptor stimulation induced reorganization of cytoskeleton proteins in rat cerebellar astrocytes. (**a**) Fluorescence images of astrocytes labelled with rhodamine-conjugated phalloidin (red) and immunostained with anti-GFAP antibodies marked with Alexa Fluor 488 (green) secondary antibodies, after time-lapse recording. Statistics show the distribution of the fluorescence intensity of phalloidin staining (**b**) and number of cells exhibiting stress fibers (**c**). Whiskers indicate SD. Statistically significant differences were calculated by employing a one-way ANOVA with Dunnett’s post-test and are indicated by * when *p* < 0.05, *** when *p* < 0.001, and **** when *p* < 0.0001.

The body stress- or adhesiveness-induced distribution observed from the mechanical mappings above open the door to a discussion on how receptor stimulation can originate a localized accumulation of anchoring points between the astrocyte and the underlying substrate, indicative of how prone to motility cells are, etc. Therefore, selective staining and observation of proteins involved in such processes might contribute to obtaining a more precise picture of the ongoing situation. P2X7R stimulation could be inducing reorganization of cytoskeleton proteins, including actin cytoskeleton [12]. Morphological changes induced by BzATP were also evidenced by staining of actin filaments with rhodamine-phalloidin (Figure 8). Treatment of astrocytes with BzATP (300 µM) altered both parameters analyzed: fluorescence intensity and cells exhibiting stress fibers. Similar to what was observed in AFM experiments, blocking of P2X7R with the antagonist A43 did not reverse BzATP effects but further increased them. Therefore, a correlation between changes in actin cytoskeleton and cell mechanical properties caused by treatment with both agonist and antagonist were observed. The increase in actin stress fibers can be associated with an increase in cell stiffness and viscosity, as shown in previous work [27,28].

## 3. Discussion

Both the presence and activity, upon nucleotide-induced stimulation, of functional P2X7 receptors in rat cerebellar astrocytes in culture were studied and have been described throughout the results reported in this manuscript. Indeed, the identification, quantification, and interpretation of cell responses upon external stimuli remain a topic of great interest for both biologists and biophysicists.

As a main difference from previous studies in the literature, the current experimental conditions and approaches are certainly innovative. For instance, in the present work, astrocytes were plated at very low density (4000 cells/cm^2^) in order to follow changes of individual, isolated, cells. This differs from biochemical studies focused on calcium imaging, on which much denser starting populations (400,000 cells/cm^2^) were needed, and samples were even incubated for over 24 h until an optimal confluence was achieved [13,14,16]. Another difference would come from the exposure time-range selected (max 4 h) to observe the P2X7R stimulation by nucleotides. For instance, previous studies covered stimulation of P2X7R in rat cerebellar astrocytes with high concentrations of BzATP over a longer period in order to show how such treatment did not induce cell death (in contrast to what was found in mouse cerebellar astrocytes) [31]. The different, and certainly critical, behavior observed between rat and mouse cells could be hypothesized to stem from differences in the density of the P2X7Rs along the plasma membrane, and/or from their coexistence with other receptors favoring cell survival (P2Y_2_R and EGFR) [16]. In general, such stimuli-induced responses have been observed to a supra-micron scale by monitoring variations on the triggered cell motility, oriented polarization, or changes in size, etc. These factors are extensively described by light-interacting microscopy techniques, from which a true quantification of the forces behind cannot be extracted, unfortunately, unless complementary approaches are considered in parallel (as is the case here).

We first analyzed the cellular behavior upon selective exposure to agonist and antagonist compounds by time-lapse microscopy. As observed in Figure 2, P2X7R stimulation with BzATP (300 µM) did not affect cell viability and increased the cell size (>20%), cell migration (>200%), and number of protrusions (>50%). In turn, the competitive P2X7R antagonist, A 438079 (or A43, as it has been referred to along the manuscript), significantly reversed the effects of BzATP treatment. These findings agree with previously reported results and highlight the connection between those changes and the mechanical properties of the cell. In order to delve into the intracellular events (i.e., cytoskeletal rearrangement) accounting for these P2X7R-induced responses, we used atomic force microscopy (AFM), which offers a well-established biophysical approach to analyze the potential changes on the mechanical properties of living rat cerebellar astrocytes on the nanometric level. These mechanical parameters were determined after stimulation or blocking of their P2X7 purinergic receptors with BzATP or A43, respectively. A sequential blocking/stimulating combination (A43 followed by exposure to BzATP), which already brought about interesting results for P2YRs to help discriminating between P2Y_1_ and P2Y_13_ receptor contributions [26], was also studied. In the current case, AFM-based studies allowed for the identification of effects beyond the mere ionotropic activity of the receptor, which are caused by alternative pathways that remain unblocked. This is here evidenced by more extreme variations in all the analyzed mechanical factors (apparent elastic modulus, adhesion force, and adhesion work) after sequential stimulation of P2X7 receptors with both A43 and BzATP, instead of the initially expected blocking. As a surprising outcome, it must be noted that trends observed for BzATP and A43 + BzATP treatments were overall the same, compared to untreated cells, except for their respective maximum adhesion force values, which went in opposite directions. This factor (F_adh_) provides an idea of the strength of the interaction between the indenting tip and the cell, which varied accordingly with the affinity (or selectivity) between both, as well as the availability shown by the cell membrane to be pulled. The latter correlates to a tighter or looser membrane–cytoskeleton connection. Cell stiffness, for instance, is among the (physical) parameters affecting the adhesive behavior of cells. As a general rule, softer samples (cells) usually induce measurement of larger adhesion events, in comparison to stiffer ones. However, in the current case, such an observation was certainly distorted. In terms of stiffness, astrocytes exposed to BzATP appeared in an intermediate level between untreated (control) cells and cells undergoing receptor pre-blocking followed by agonist stimulation (A43 + BzATP). This would lead to predicting a gradual decrease in F_adh_ from one case to the other. Surprisingly, our measurements suggest a generation of stickier cells upon direct exposure of P2X7 to BzATP in comparison to those generated after A43 blocking. Divagation over this point may be rather diverse: the membrane–cytoskeleton connection was not as strengthened as actin fibers (and other constitutive elements) within the cytoskeleton were (see explanation below); the surface charge was locally affected by means of the activation performed; or, perhaps, a different type of process was triggered. Indeed, a very similar trend was observed in our previous work—when testing exposure to UTP to identify the presence of P2Y_2_ receptors in astrocytes. P2Y2R is one of the receptors inducing cell migration, and its stimulation also resulted in stiffer cells, albeit more adhesive [26].

AFM was also used to determine the viscoelastic behavior of the astrocytes. A good representative of this behavior is the cell viscosity value derived from cytoskeleton contribution, which increased up to a 150% after direct receptor simulation with BzATP when compared with the pre-blocked case (1171.2 vs. 1775.0 Pa s, BzATP and A43 + BzATP, respectively). A larger viscosity clearly pointed towards re-arrangement of cytoskeleton constituents to form a much-reinforced structural supportive mesh. This also correlated with the detection of a stiffer cell when P2X7Rs were initially passivated prior to stimulation, attending to the instantaneous elastic modulus (2.00 vs. 3.06 kPa) behavior. Such reinforcement seemed not to have a dramatic impact at the so-considered cortex level, since $\eta_{1}$ did not vary much, despite an increment of the 58% between A43 + BzATP-treated cells and the control system that should be noted. Additionally, astrocytes treated with BzATP showed more actin stress fibers compared to control conditions (Figure 8), therefore confirming the implication of actin cytoskeleton in the increase in viscosity observed after P2X7 stimulation.

Finally, force spectroscopy maps of the astrocytes (see Figure 6 and Figure 7) were generated for the first time. These maps clearly indicated that the cell response upon receptor stimulation occurred all over the cell body, with a special mention to what was observed for adhesion under direct treatment with BzATP: a huge general increase in attachment independently of the cell region, which was not observed under receptor pre-blocking. This result brings us to the discussion above, again showing certain similarities between the effects of P2X7R and P2Y_2_R stimulation. In rat cerebellar astrocytes, both P2X7 and P2Y_2_ receptors increase intracellular calcium levels and share several intracellular pathways, including protein kinase D (PKD), and mitogen activated protein kinases (MAPKs), such as the extracellular regulated kinases 1 and 2 (ERK1/2) [14,15,16] and p38 [32]. Furthermore, both receptors also induce transactivation of epidermal growth factor receptor (EGFR), which is essential for the inactivation of MAPKs, preventing MAPK overactivation that would lead to cell death (unpublished results). Despite sharing several intracellular pathways, stimulation of each receptor causes different effects on astrocytes. As mentioned above, P2Y_2_ stimulation is linked to astrocyte migration [16], while P2X7 receptor activation causes morphological changes [17]. Surprisingly, in the current work, we also determined a slight increment in the migration of astrocytes after P2X7 receptor stimulation. Thus, such an effect could be, at least in part, responsible for the similarity in the changes observed in the mechanical properties of the astrocytes after stimulation of both receptors (stiffer and more adhesive cells). In addition, the activation of MAPKs has been previously linked to actin cortex reorganization in other cell models [12]. Since MAPKs are activated by both receptors, their implication in the reorganization of the actin cytoskeleton in astrocytes cannot be discarded. Further experiments, employing inhibitors of different MAPKs, are required to determine the effects of this pathways in our cell model.

A final surprising result to mention from the present work is the different effects of the P2X7R antagonist, A43, on both the mechanical properties of the astrocytes and on their activity. A43 completely abolished the increase in migratory capabilities, cell spreading, and membrane protrusions of astrocytes treated with BzATP. However, when analyzing the mechanical properties of the astrocytes with AFM, A43 did not hinder the effects of BzATP on the stiffness and viscosity of the cells. In fact, it appeared to enhance such effects instead. The same applies for the effects observed on the actin cytoskeleton. Such inconsistency in the effects of A43 may lie on the distinctive features of P2X7 receptors. As indicated previously, P2X7 receptors possess the largest C-termini present in P2X receptors. Such a particular feature allows P2X7 receptors to interact with different proteins, such as integrins and β-actin [11], and also to activate different signaling pathways, such as phospholipase C (PLC), protein kinase C (PKC), and phosphatidil-inositol-3 kinase (PI3K), which are independent from ion channel activation (for a review, see [10]). While A43 may block the activity of the ionic channel and calcium influx, this type of signaling activated by the C-termini is independent and therefore unaffected by A43 activity. On one hand, the increase on the migratory capabilities of astrocytes after P2X7 stimulation could be dependent on channel ionic activity, and therefore blocked by A43; on the other hand, further effects on the actin cytoskeleton and, by extension, on the mechanical properties of the cell, could be dependent of the activity of the C-termini, and as such, unaffected by A43. Previous work of the group, related to the activation of ERK1/2 in astrocytes after P2X7R stimulation, supports this hypothesis [32]. Treatment with A43 prior to stimulation of the receptors with BzATP alters the temporal pattern of phosphorylation and activation of ERK1/2, but it does not completely block it [32]. Although implication of the C-termini in this process is not confirmed, it could explain the activation of ERK1/2 despite the treatment with the antagonist, possibly by means of EGF receptor transactivation [16,32]. As stated above, MAPKs, including ERK1/2, are linked to actin cytoskeleton remodeling, and since A43 does not block the activation of these MAPKs after P2X7 stimulation, this could also explain the inability of the antagonist to block the changes in actin cytoskeleton and the mechanical properties of the cell. The enhancement of the effects observed after treatment with both A43 and BzATP, however, suggests complex interactions between different signaling pathways. Unraveling such complex effects elicited by P2X7R stimulation, however, requires further research and is out of the scope of the current work.

## 4. Materials and Methods

### 4.1. Chemicals, Materials, and Antibodies

Papain was purchased from Worthington (Lake Wood, NJ, USA). Fetal bovine serum (FBS), Dulbecco’s modified Eagle’s medium (DMEM), penicillin/streptomycin solution, Leibovitz’s-L15 and TrypLE™ Express, rhodamine-conjugated phalloidin, and nuclear counterstain DAPI (4′,6-Diamidino-2-phenylindole) were obtained from Gibco, Thermo-Fisher (Waltham, MA, USA). Plastic Petri dishes and culture flasks were supplied by Falcon Becton Dickinson Labware (Franklin Lakes, NJ, USA). Borosilicate circular cover glasses (diameter: 24 mm, 15 mm, thickness: 0.08–0.12 mm) were purchased from Menzel Gläser, VWR, (Bruchsal, Germany). Bovine serum albumin (BSA), BzATP (2′(3′)-O-(4-benzoylbenzoyl)adenosine 5′-triphosphate), A438079 (3-(5-(2,3-dichlorophenyl)-1H-tetrazol-1-yl)methyl pyridine), and anti-GFAP antibody were provided by Sigma-Aldrich, Merck (Kenilworth, NJ, USA). Alexa Fluor 488 secondary antibody was from Dako/Agilent (Santa Clara, CA, USA). All other non-specified reagents were routinely supplied by Sigma-Aldrich, Merck, or Roche Diagnostics SL (Basel, Switzerland).

### 4.2. Cell Cultures

Primary cultures of cerebellar astrocytes were prepared at the Complutense University of Madrid, following the International Council for Laboratory Animal Science guidelines, as described previously [15]. Cerebella from Wistar rat pups (P7) were digested with papain; the resulting cells were resuspended in complete DMEM medium (10% (*v/v*) FBS, 2 mM glutamine, 25 mM glucose, 100 U/mL penicillin, and 100 mg/mL streptomycin) and then seeded in culture flasks at a density of 70,000 cells/cm^2^. The cells were maintained in culture until they reached confluence, and then they were purified by orbital shaking. Purified astrocytes were then detached from the culture flasks by trypsin digestion and cryopreserved in FBS containing 10% (*v/v*) DMSO at −80 °C. Cryopreserved cells were thawed when necessary, resuspended in complete culture medium, and grown in flasks until reaching confluence again.

### 4.3. Time-Lapse Video Microscopy, Single-Cell Tracking, and Immunofluorescent Staining

24 h before time-lapse video microscopy experiments, astrocytes were seeded at very low density (4000 cells/cm^2^) onto 15 mm coverslips in 24-well plates in complete culture medium. Before the experiment, cells were serum-starved for 30 min in complete DMEM medium containing only 1% FBS and then stimulated with 300 µM BzATP for 1 h. Where indicated, cells were pretreated with P2X7 antagonist (A 438079, 10 µM) for 5 min, prior to the nucleotide addition.

After treatment, cells were tracked using a NIKON TE-2000 microscope and equipped with a long-distance 20x phase contrast objective (Nikon) and a ZYLA camera from ANDOR [33]. Cells were kept at a constant temperature of 37 °C and in 5% CO_2_, and images were acquired every 5 min over 3 h using the 4.7/NIS-elements software from NIKON.

After finishing the time-lapse experiments, cells were fixed with 4% (*w/v*) paraformaldehyde for 10 min at room temperature, followed by cell permeabilization with 0.1% (*v/v*) Triton X-100 in phosphate-buffered saline (PBS) for 15 min at room temperature. Unspecific antibody interactions were blocked by adding 2% (*w/v*) bovine serum albumin (BSA) in PBS for 1 h at room temperature, and then the samples were incubated with a primary anti-GFAP rabbit antibody and diluted 1:1000 in 1% (*w/v*) BSA–PBS solution overnight at 4 °C. This was followed by incubation for 1 h at room temperature with a 1% (*w/v*) solution of BSA–PBS containing a secondary goat anti-rabbit antibody labelled with the fluorophore Alexa Fluor™ 488, diluted 1:500; a rhodamine-labelled phalloidin actin marker, diluted 1:40; and a nuclear counterstain DAPI, with a final concentration of 2 µg/mL. After a final washing step with PBS, samples were air-dried, covered in mounting medium, and stored at 4 °C until their visualization in the fluorescence microscope.

Cell mobility was determined by single-cell tracking, using specific custom software (The Tracking Tool, TTT) [34]. Migrated distance was determined as the total distance covered by each cell during the 3 h of time-lapse, on the basis of the displacement of the cell nucleus.

### 4.4. Atomic Force Microscopy

Before AFM experiments (48 h in advance), astrocytes were plated at very low density (4000 cells/cm^2^) onto 24 mm diameter glass coverslips (previously cleaned with oxygen plasma) in 6-well plates in complete culture medium. Right before the experiments, cells were subjected to trophic factor withdrawal by washing them for 30 min with Leibovitz’s medium, and then the cells were stimulated with 300 µM BzATP in the same medium for 1 h at 37 °C. Where indicated, cells were pretreated with P2X7 antagonist (A 438079, 10 µM) for 5 min, prior to the nucleotide addition.

AFM measurements were carried out in a JPK Nanowizard III (JPK Instruments, Berlin, Germany), mounted on an inverted optical microscope (Axio Observer Z1, Zeiss, Jena, Germany), and equipped with a CellHesion module and a BioCell chamber (JPK Instruments, Berlin, Germany). To ensure the duration and quality of the experiments, cells were maintained in Leibovitz’s medium at 37 °C. Before each experiment, triangular silicon nitride cantilevers equipped with a quadratic pyramidal tip (DNP-10, B, Bruker; r = 22 nm, α = half-to-face-angle = 22°) were cleaned with ozone/UV and calibrated on a clean glass surface covered in Leibovitz’s medium at 37 °C, and their spring constant was determined by means of the thermal tune method (nominal spring constant of 0.12 N/m).

Force spectroscopy curves were obtained as previously described [21,26,35]. Cells were indented on top of the nucleus to reduce variability and influence of the substrate on the measurements. The tip was approached at a constant velocity of 5 µm/s, and after reaching a setpoint of 1.5 nN, it was kept in contact at constant height (stress–relaxation measurements) for a period of 5 s. The tip was then retracted at the same constant velocity (5 µm/s). To ensure tip cleanliness, the glass substrate was probed in between the indentation of cells. For statistical purposes, each cell was indented 5 times, and at least 20 cells per condition were analyzed.

Complementary mapping analysis was applied on individual cells, after the selection of an optimal zone and performance of an optical calibration. Then, measurements were run over a 32 × 32 pixel map covering between 30 and 40 µm^2^ areas, attending to the respective cell shapes. Each of these pixels originated individual force vs. distance plots recorded at the same conditions described above. As a main difference to previous measurements, the pause segment was omitted in the recording of the maps (retraction started at maximum load achievement) due to experimental feasibility, and the tip was immediately retracted at maximum loading force.

### 4.5. AFM Data Analysis

Force curves were analyzed using JPKSPM Data Processing software (JPK Instruments, Berlin, Germany) and the R afmToolkit [36]. The batch data processing from JPK was particularly useful in the case of force-volume maps and also offered a good starting point for comparison with those analyzed with R in the case of individual measurements. Regarding R afmToolkit, force spectroscopy curves were imported into the toolkit, and contact point detection, baseline calibration, and zero-point determination were realized using the corresponding algorithms of the toolkit [37]. Deformation of the sample ($\delta_{s}$) was determined as indicated in Equation (1):

(1) $$\delta_{s}=Z_{p}-\delta_{c}=Z_{p}-\frac{F}{k_{c}},$$

where $Z_{p}$ is the extension of the piezo, $\delta_{c}$ is the deformation of the cantilever, F is the force, and $k_{c}$ is the spring constant of the cantilever. Apparent Young’s modulus ($E_{app}$) values were obtained for an indentation range of 500 nm, following a Hertz–Sneddon model optimized for the tip geometry employed (quadratic pyramid) according to Equation (2):

(2) $$F=\frac{E_{app}}{1-\nu }\frac{\tan\left(\alpha \right)}{\sqrt{2}}\delta^{2},$$

where F is the force, ν is the Poisson ratio (set to 0.5), α is the half-to-face angle of the tip used (22°), and δ is the indentation of the sample.

Stress–relaxation pause segments were used to determine the viscoelastic properties of the cell. A double exponential decay function was fitted to the stress–relaxation segments, and calculation of the different viscoelastic values were determined by applying a Zener model of the cell (a spring in parallel with two Maxwell elements), following Equation (3):

(3) $$F\left(t\right)=\frac{C}{1-\nu }\left[E_{\infty }+\overset{2}{\underset{i=1}{\sum }}E_{i}\exp\left(-\frac{t}{\tau_{i}}\right)\right],$$

where ν is the Poisson ratio, $E_{\infty }$ is the equilibrium modulus (at infinite time), $E_{i}$ is the compressive moduli of the respective springs of the Maxwell elements, $\tau_{i}$ is the relaxation times of the viscous dashpots, and *C* is a constant that depends on the geometry of the indenter. For pyramidal tips, *C* is defined as indicated in Equation (4):

(4) $$C_{pyramidal}=\frac{1}{\sqrt{2}}\tan\alpha \delta_{0}{}^{2},$$

where α is the half-to-face angle of the tip used and $\delta_{0}$ is the indentation value that is kept constant during the stress relaxation phase. Lastly, viscosities of the dashpots ($\eta_{i}$) were calculated following Equation (5):

(5) $$\eta_{i}=E_{i}\tau_{i},$$

where $E_{i}$ is the compressive moduli of the respective springs of the Maxwell elements and $\tau_{i}$ is the relaxation times of the viscous dashpots.

Retract curves were adapted similarly, and maximum adhesion force was measured as the lowest point of the curve in the Y-axis. The total number of rupture events, their relative position, and the rupture force associated were also saved for further analysis.

In the case of colored maps, these were obtained after batch processing of the related plots, data exporting of the thus extracted factors, and final plotting in Origin software.

### 4.6. Statistical Analysis and Data Presentation

Statistical analysis of AFM data was carried out using the OriginPro 2018 (OriginLab, Northhampton, MA, USA) program. At least 20 cells per experimental condition were analyzed, and 5 force curves were obtained from each cell. Values for the apparent Young’s modulus ($E_{app}$), cell indentation, maximum adhesive force, compressive moduli, relaxation times, and viscous moduli were obtained as indicated above, and the outliers were removed using Grubb’s test. Statistically significant differences between the different experimental conditions were determined with an analysis of the variance test (one-way ANOVA) followed by Dunnett’s post-test analysis, and they are reported in the figures as * for *p* < 0.05, ** for *p* < 0.01, *** for *p* < 0.001, and **** for *p* < 0.0001. Data graphs were generated in OriginPro 2018 and further processed using Adobe Illustrator Adobe Inc, San José, CA, USA.

Statistical analysis of time-lapse video microscopy experiments was performed using GraphPadPrism8 (GraphPadsoftware), and one-way ANOVA followed by Dunnett’s multiple comparisons tests was used to analyze the results. The data are presented as the mean ± SD, and each independent experiment shown was reproduced at least three times. A *p*-value < 0.05 was considered statistically significant.

## 5. Conclusions

The present work demonstrates yet another role of P2X7 receptors in the vast array of functions inside the cell. Stimulation of P2X7 receptors increase the migratory capabilities, spreading and membrane protrusions of rat cerebellar astrocytes seeded at low density. Analysis of the mechanical properties of the cell with atomic force microscopy indicate an increase in cell stiffness, viscosity, and cell adhesion—the former two can be related to the increment observed in actin stress fibers. Surprisingly, treatment with the antagonist of P2X7 receptor A43, prior to P2X7 stimulation, only blocks the increment in cell migration, spreading, and membrane protrusions; the mechanical properties of the cell, as well as the presence of actin stress fibers, are further enhanced by this. This contradictory effect indicates that P2X7R is not a simple ionic channel but a complex receptor, capable of activating different signaling pathways that are independent from the channel activity.

## Figures and Tables

**Table 1:** Statistics on data depicted in Figure 2 and Figure 3 obtained after 3 h of time-lapse video microscopy. Values represent the means and the standard deviation (SD). A pixel corresponds to 0.5 µm.

	Cell Area (µm^2^)	Membrane Protrusions/Cell	Migration (Pixel)
Control (untreated)	812 ± 138	3.10 ± 0.70	33.00 ± 35.14
BzATP	1001 ± 215	4.85 ± 1.28	78.90 ± 49.81
A43 + BzATP	765 ± 166	3.96 ± 1.17	27.04 ± 20.33

**Table 2:** Statistics on approach segment (mean indentation and apparent elastic modulus). Error values indicate the standard deviation (SD).

System (1 h Incubation)	Indentation (µm) ± SD	E_app_ (kPa) ± SD
Control (untreated)	1.07 ± 0.53	4.84 ± 4.69
BzATP	0.82 ± 0.33	5.93 ± 4.42
A43	0.99 ± 0.42	4.03 ± 2.70
A43 + BzATP	0.81 ± 0.52	8.60 ± 7.76

**Table 3:** Statistics on pause segment (mean viscous moduli, $\eta_{1}$ and $\eta_{2}$). Error values indicate the standard deviation (SD).

System (1 h Incubation)	$\eta_{1}\;(\mathrm{Pa}\;s)\;\text{±}\;\mathrm{SD}$	$\eta_{2}\;(\mathrm{Pa}\;s)\;\text{±}\;\mathrm{SD}$
Control	31.9 ± 22.9	590.8 ± 510.1
BzATP	44.9 ± 29.2	1171.2 ± 1095.0
A43	31.8 ± 16.7	680.1 ± 523.8
A43 + BzATP	50.4 ± 45.2	1775.0 ± 2231.5

## Supplementary Materials

The following supporting information can be downloaded at: https://www.mdpi.com/article/10.3390/ijms231911927/s1.
