# Supplementary material for: Nucleotide-Induced Nanoscale Changes in the Mechanical Properties of Rat Cerebellar Astrocytes: Selective Stimulation and Blocking of the Purinergic Receptor P2X7
Source: Int J Mol Sci. 2022 Oct 7;23(19):11927. doi: 10.3390/ijms231911927 (PMC9570466; doi:10.3390/ijms231911927)
Supplement: Supplementary file 1 [file ijms-23-11927-s001.zip › ijms-1941302-supplementary.pdf]

Table S1. Statistics on retract segment: maximum adhesion force (F_{adh}), maximum adhesion force peak position and work of detaching (W_{adh}). Error values indicate the SD.

System (1h Incubation)	F_{adh} (pN) ± SD	Peak Position (μm) ± SD	W_{adh} ($x10^{-16}$, J) ± SD
Control	194.9 ± 61.2	0.78 ± 0.36	3.79 ± 3.18
BzATP	213.2 ± 45.0	0.73 ± 0.23	2.89 ± 2.30
A43	176.8 ± 47.4	0.85 ± 0.39	3.02 ± 2.68
A43 + BzATP	142.0 ± 30.0	0.71 ± 0.25	1.86 ± 1.87

Table S2. Statistics on pause segment (compressive moduli). Error values indicate the SD.

System (1 h Incubation)	E_∞ (kPa) ± SD	E_1 (kPa) ± SD	E_2 (kPa) ± SD	E_{inst} (kPa) ± SD
Control	0.43 ± 0.31	0.31 ± 0.18	0.29 ± 0.20	1.09 ± 0.79
BzATP	0.90 ± 0.70	0.52 ± 0.34	0.46 ± 0.32	2.00 ± 1.54
A43	0.65 ± 0.45	0.42 ± 0.26	0.32 ± 0.20	1.30 ± 0.73
A43 + BzATP	1.52 ± 1.65	0.59 ± 0.56	0.69 ± 0.75	3.06 ± 3.47

Table S3. Statistics on pause segment (relaxation times). Error values indicate the SD.

System (1 h Incubation)	τ_1 (s) ± SD	τ_2 (s) ± SD
Control	0.097 ± 0.049	2.097 ± 0.966
BzATP	0.093 ± 0.046	2.214 ± 0.917
A43	0.087 ± 0.037	2.104 ± 0.831
A43 + BzATP	0.088 ± 0.041	2.330 ± 0.940

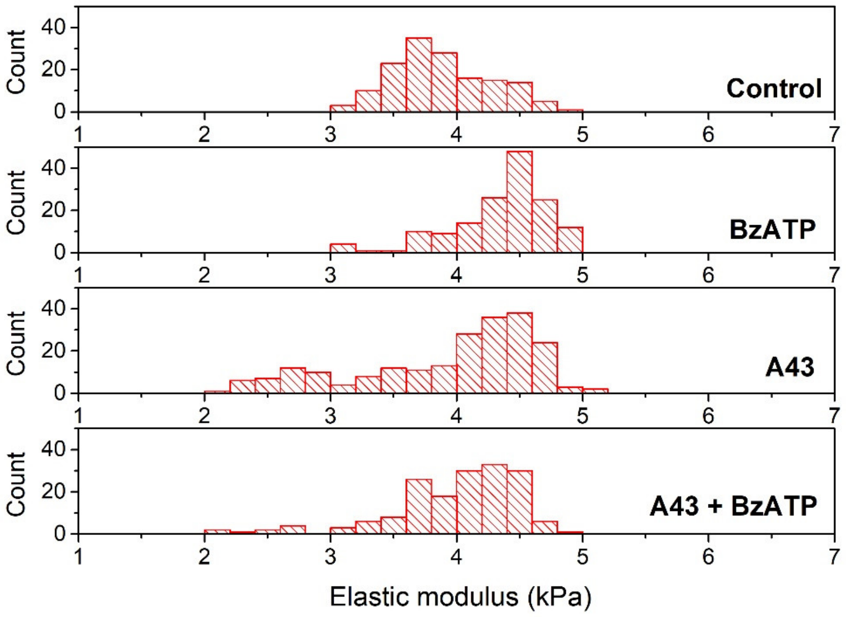

Figure S1. Elastic Moduli (log)histogram comparison from astrocyte force mappings (Figure 6) under the indicated conditions.

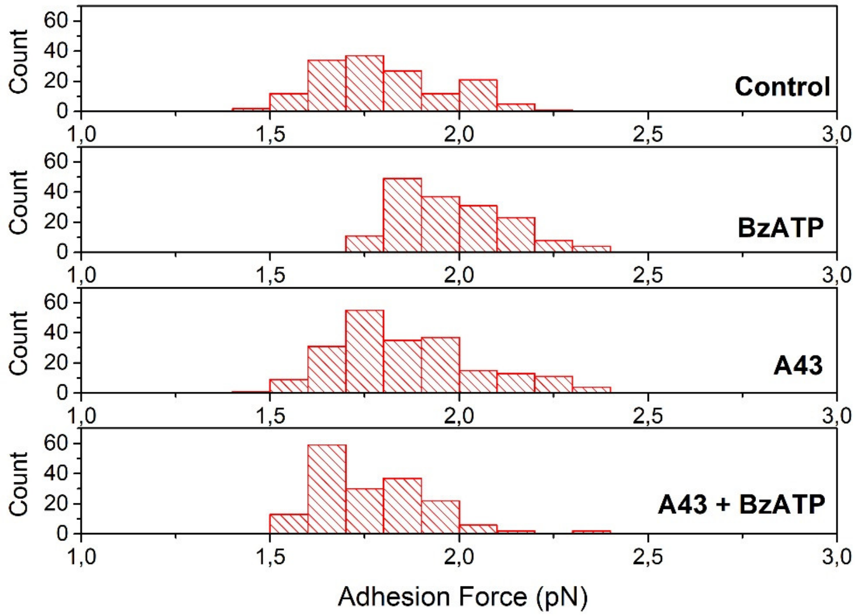

Figure S2. Adhesion Force (log)histogram comparison from astrocyte force mappings (Figure 7) under the indicated conditions.
